# Psychometric validation of the Patient Health Questionnaire-9 in Chinese adolescent and adult psychiatric inpatient populations

**DOI:** 10.3389/fpsyt.2025.1657696

**Published:** 2025-10-29

**Authors:** Wei Li, Jia-Yi Yin, Qian Wang, Jie Zhong

**Affiliations:** ^1^ Shanxi Bethune Hospital, Shanxi Academy of Medical Sciences, Third Hospital of Shanxi Medical University, Tongji Shanxi Hospital, Taiyuan, China; ^2^ Beijing Key Laboratory of Behavior and Mental Health, Clinical and Health Psychology Department, School of Psychological and Cognitive Sciences, Peking University, Beijing, China; ^3^ Department of Psychology, School of Humanities, Tongji University, Shanghai, China

**Keywords:** Patient Health Questionnaire-9 (PHQ-9), depressive disorder, Chinese psychiatric inpatients, adolescent, validity

## Abstract

**Background:**

Depressive disorder represents a major public health burden globally, yet the validity of the Patient Health Questionnaire-9 (PHQ-9)—a widely used depression screening tool—remains underexplored in Chinese psychiatric inpatient populations, particularly in age-stratified analyses. This study aimed to (1) validate the Chinese version of the PHQ-9 in Chinese psychiatric inpatients (contrasting with community-based findings) and (2) compare its psychometric properties between adolescent and adult inpatients.

**Methods:**

This cross-sectional study enrolled 485 psychiatric inpatients (including 105 adolescents) from Shanxi Bethune Hospital. Participants completed the Chinese version of the PHQ-9. Analyses encompassed confirmatory factor analysis (CFA), Gaussian Graphical Model-based network analysis, and receiver operating characteristic (ROC) curve analysis to determine optimal diagnostic cutoff scores.

**Results:**

Results showed the PHQ-9 had good internal consistency: Cronbach’s α = 0.876 (adolescents) and 0.883 (adults). CFA revealed no significant difference in fit between the unidimensional and two-factor (cognitive-affective vs. somatic) models in adolescents (Δχ²=0.79, p=0.374), with both models showing marginal fit (likely affected by small sample size). In adults, the two-factor model was preferred (Δχ²=6.49, p=0.011). The Network Comparison Test found no significant differences in network structure (M = 0.211, p=0.598) or global strength (S = 0.262, p=0.186) between age groups, but the adolescent network had poor stability (correlation stability coefficient = 0), limiting interpretation. ROC analysis identified age-specific optimal cutoffs exceeding the conventional threshold of 10: 15.5 for adolescents (sensitivity=0.84, specificity=0.47) and 14.5 for adults (sensitivity=0.79, specificity=0.66). Notably, 64.7% of the total sample scored ≥15 on the PHQ-9, while only 43.7% had a primary diagnosis of depressive disorder (ICD-11 6A7), indicating comorbid depressive symptoms contributed to higher cutoffs.

**Conclusion:**

The findings of this study validate the structural validity and diagnostic validity of the PHQ-9 among Chinese adult psychiatric inpatients, while emphasizing that the interpretation of its factor structure in the adolescent population requires caution. The age-related symptom topological patterns indicated by network analyses are highly likely to be influenced by the insufficient size of the adolescent sample and need to be confirmed by subsequent studies. The results of the ROC curve highlight the clinical significance of formulating population-specific diagnostic cutoffs; however, the impact of comorbidity on the findings of this study must be taken into consideration.

## 1 Introduction

Depressive disorder (hereafter referred to as depression) is a highly prevalent mental health condition. The World Health Organization (1) notes that it affects approximately 3.8% of the global population and that over 700,000 suicide deaths occur annually and are attributable to this disorder. Effective screening and diagnosis are critical to identifying individuals requiring intervention. The 9-item Patient Health Questionnaire (PHQ-9) stands as one of the most widely validated and utilized depression screening instruments in both research and clinical contexts (2). Derived from the Primary Care Evaluation of Mental Disorders (PRIME-MD; 3), which was originally anchored in DSM-III-R and DSM-IV diagnostic criteria, the PHQ-9 operationalizes these criteria via a brief self-report format (3, 4). Comprising nine items rated on a 4-point Likert scale, the PHQ-9 assesses the frequency of affective, cognitive, and somatic depressive symptoms over a two-week period. A cutoff score of 10 is conventionally used to denote clinically significant depression, demonstrating 88% sensitivity for major depressive disorder and 93% specificity for non-depressed individuals (4). Subsequent meta-analytic evidence (5, 6) has confirmed that this threshold optimizes the balance between sensitivity and specificity.

The PHQ-9 has exhibited robust psychometric properties across multicultural samples and clinical contexts (4, 7, 8), with cumulative evidence supporting its measurement invariance across linguistic, cultural backgrounds, gender, and age-related dimensions.

In Chinese-speaking populations, the PHQ-9 has also been extensively employed (9), with prior investigations confirming its psychometric stability in diverse samples, including adolescents (10), university students (11), general hospital inpatients (12), and psychiatric patients diagnosed with depression (13, 14). Notwithstanding, the majority of these studies have focused on community samples or diagnostic subgroups, often treating adolescents and adults as homogeneous populations or independent cohorts. Given the increasing recognition of age-related heterogeneity in depression symptomatology (15), whether the PHQ-9 maintains consistent structural and clinical properties across adolescent and adult psychiatric inpatients remains unaddressed—a critical gap for refining age-specific diagnostic protocols. Given the developmental disparities in symptom presentation and clinical needs, a comprehensive psychometric evaluation of the PHQ-9 across these age groups is essential to clarify its structural validity and inform evidence-based screening practices in psychiatric settings.

The factor structure of the PHQ-9 remains a topic of enduring scientific debate (16). Central to this discourse is the controversy regarding whether the scale is best conceptualized as unidimensional or two-factor, with additional heterogeneity in item allocation within two-factor models. Accumulating empirical evidence supports a unidimensional structure: Shevlin et al. (17) demonstrated optimal fit of a single-factor model across four European populations, and Bianchi et al. (18) reported congruent findings in a large community sample. Two-factor models typically aim to discriminate between cognitive/emotional and somatic symptom domains; conversely, studies such as Shin et al. (19) proposed alternative structures, including an “affective-somatic”/”cognitive” two-factor model in a Korean general population. A comprehensive review by Lamela et al. (16) concluded that although two-factor models occasionally exhibit marginally better model fit, unidimensional solutions are supported by more consistent cross-sample evidence. Notably, researchers (18, 20) have highlighted that even in studies favoring two-factor solutions, substantial cross-sample variability in item loadings and high inter-factor correlations challenge the discriminant validity of these factors.

Given the established influence of cultural contexts on the conceptualization and expression of mental health symptoms (21), the factor structure of the PHQ-9 is hypothesized to exhibit population-level variability. In a cross-cultural study of antenatal women across eight low-resource countries, a unidimensional model provided the optimal fit in four populations, while a cognitive-motivational/somatic two-factor model demonstrated superior fit in Jamaica. Notably, no optimal model fit was achieved in the remaining three countries. Network analysis within the same study revealed that although the pattern of associations between PHQ-9 items was consistent across countries—and correlations with external measures aligned with theoretical expectations (22)—the magnitude of these associations varied significantly.

Collectively, these findings suggest that no single factor model can be universally optimal across cultural and clinical contexts; instead, factor structural solutions should be evaluated and validated within specific populations. In the Chinese context, Sun et al. (14) demonstrated that a unidimensional model provided the best fit for data from hospitalized and outpatient patients with depression, while Feng et al. (13) reported congruent results in a sample of depressed inpatients. However, the generalizability and robustness of these findings across broader psychiatric inpatient populations—encompassing diverse diagnostic categories—remain underexplored, creating a critical gap with implications for the diagnostic process.

This gap is further widened by two key limitations in existing research on the PHQ-9. First, the majority of studies have focused on community samples or narrow diagnostic subgroups, leaving the measure’s validity in the aforementioned broader psychiatric inpatient populations (with diverse diagnoses) largely unaddressed. Second, even in inpatient-focused research, adolescents and adults are often treated as a homogeneous group, despite growing evidence of age-related heterogeneity in depression symptomatology (23). To address these dual shortcomings, the present study aims to: (1) validate the Chinese version of the PHQ-9 specifically in Chinese psychiatric inpatients—a focus that stands in critical contrast to community-based findings (e.g., 11, who reported a conventional cutoff of 10 for general populations); and (2) compare the measure’s psychometric properties (including factor structure, network characteristics, and diagnostic cutoffs) between adolescent and adult inpatients. This dual objective not only fills the inpatient-specific validity gap identified earlier but also explores age-related differences within this clinical population, addressing a long-overlooked dimension of PHQ-9 validation.

## 2 Method

### 2.1 Subjects and procedure

All eligible psychiatric inpatients (n = 552) admitted to Shanxi Bethune Hospital between September 1, 2023, and September 22, 2024, were invited to participate in this study. The inclusion criteria were defined as follows: (1) aged ≥9 years (the minimum age for self-reporting PHQ-9 items in Chinese clinical settings, as established by Wang et al. (24); (2) admitted to the psychiatric inpatient unit of Shanxi Bethune Hospital; (3) capacity to complete self-report questionnaires (confirmed by attending psychiatrists, with no severe cognitive impairment or language barriers); and (4) provision of informed consent (with consent obtained from parents/guardians for participants ≤16 years old). The exclusion criteria included: (1) diagnosis of severe neurocognitive disorders (e.g., Alzheimer’s disease); (2) acute psychotic episodes requiring emergency intervention; and (3) refusal to participate.

The PHQ-9 was administered before 24 hours of admission to capture baseline symptoms. 85 participants (17.9%, 85/485) were receiving pharmacological treatment (antidepressants, mood stabilizers, or antipsychotics) before the time of assessment. ICD-11 diagnoses were made by board-certified psychiatrists (≥5 years of experience) during admission evaluations (within 24 hours of entry).

After the application of predefined inclusion and exclusion criteria, a total of 519 patients (including 111 adolescents aged < 16 years) successfully completed the entire study participation process. Among these 519 participants, 485 individuals completed the Patient Health Questionnaire-9 (PHQ-9) in full, with no missing data observed in their responses. Of the patients who finished the PHQ-9, 105 were adolescents aged < 16 years. Based on the total number of eligible individuals initially invited to participate in this study, the response rate was calculated to be 93.4%.

Listwise deletion was applied to participants with missing PHQ-9 responses, resulting in a final analytical sample of 485 individuals (including 105 adolescent inpatients aged < 16 years). To assess the randomness of missing PHQ-9 data, a re-conducted Little’s Missing Completely at Random (MCAR) test was performed. With the revised sample structure (485 valid cases and 34 cases excluded due to missing PHQ-9 responses), the test yielded a chi-square statistic (χ²) of 0.302 with 2 degrees of freedom (df = 2) and a non-significant p-value (p > 0.05). This result confirms that the missingness of PHQ-9 data remained random, thereby justifying the use of listwise deletion (25, 26). The proportion of excluded cases was low (6.55%, 34/519). Additionally, sensitivity analyses using multiple imputation consistently replicated the key findings, and detailed results of these analyses are available upon request.

The selection of the 16-year age cutoff was based on three considerations: (1) compliance with Chinese psychiatric inpatient protocols, which mandate parental consent for patients <16 years old and specify management in adolescent-specific units; (2) consistency with previous validation studies of the PHQ-9 among Chinese adolescent inpatients (13); and (3) alignment with developmental capacity for independent self-reporting (10). It is noted that this age cutoff reflects clinical practice rather than the World Health Organization’s (WHO) general definition of adolescence (10–19 years old).

Among participants aged 9–11 (n = 32), the PHQ-9 was administered with two specific adaptations: (1) Trained research assistants provided simple rephrasings of items to ensure age-appropriate comprehension—for instance, the term “anhedonia” was rephrased as “not enjoying things you used to like”; (2) Post-completion comprehension checks were conducted to confirm that participants had understood each item. These adaptations align with the prior validation of the PHQ-9 for young Chinese adolescents, which was derived from the work of a collaborating research team (27). Other participants completed self-report questionnaires and received clinical diagnoses from psychiatrists based on ICD-11 criteria. Data confidentiality was ensured, with no personal information disclosed without informed consent. The study was approved by the Ethics Committee of Peking University. Written informed consent was obtained from all participants, or from parents/guardians for individuals under the age of 16.

### 2.2 Materials

The 9-item Patient Health Questionnaire (PHQ-9) is a self-administered instrument comprising nine items to evaluate depressive mood, cognitive, and somatic symptoms over the past two weeks. Items cover domains including anhedonia, depressed affect, sleep disturbance, fatigue, appetite changes, low self-esteem, concentration difficulties, psychomotor alterations, and suicidal ideation. Each item is rated on a 4-point Likert scale: 0 (not at all), 1 (on several days), 2 (on more than half the days), and 3 (nearly every day) (4).

The Chinese version of the PHQ-9 has demonstrated robust reliability and validity in adolescents, including those aged 9–16. Leung et al. (10) reported α=0.85 and a unidimensional structure in 12–17-year-olds, while Wang et al. (24) confirmed α=0.82 and convergent validity with clinician ratings in 9–11-year-olds (with minimal caregiver assistance for wording clarification). This protocol was adopted for participants <12 years in the current study.

### 2.3 Analysis

All statistical analyses were performed using R version 4.2.3, encompassing descriptive statistics, internal consistency evaluation (Cronbach’s alpha), confirmatory factor analysis (CFA), receiver operating characteristic (ROC) analysis, and network analysis. The CFA was employed to explore the latent structural validity of the PHQ-9 in both adolescent and adult subgroups. ROC analysis was conducted to assess the diagnostic accuracy of the PHQ-9, with depressive disorder defined by ICD-11 criteria (6A70) serving as the gold standard. For network analysis, Gaussian Graphical Models (GGMs) were separately estimated for adolescents and adults using the EBICglasso algorithm implemented in the bootnet R package (28). In this framework, nodes represented individual PHQ-9 symptoms, while edges denoted partial correlations between symptoms after controlling for all other variables. Centrality indices (strength, closeness, betweenness, and expected influence) were calculated to identify pivotal symptoms. The stability and accuracy of network estimates were evaluated via bootstrapped confidence intervals and correlation stability coefficients. Group differences in network structure and global strength were compared using the Network Comparison Test (29).

## 3 Result

### 3.1 Descriptive statistics

The mean age of the 485 valid patients was 35.96 years (*SD* = 20.03), with ages ranging from 9 to 81 years. The age distribution was as follows: median = 32 years (IQR = 17–48 years); age bands: 9–15 years (adolescents, 105/485, 21.6%), 16–30 years (142/485, 29.3%), 31–50 years (158/485, 32.6%), and 51–81 years (80/485, 16.5%). This confirms that adolescents constitute a distinct subgroup, while adults are concentrated in the 16–50 age range (61.9%), consistent with the typical age profile of Chinese psychiatric inpatients (13). The majority of patients were female. The most prevalent diagnoses were depressive disorders and anxiety- or fear-related disorders. Patients were classified into two age groups: adolescents (under 16 years) and adults (16 years and above). Detailed demographic data are presented in **Table 1**. Patients were further categorized according to their ICD-11 diagnoses and PHQ-9 scores.

**Table 1:** Demographic data and descriptive statistics.

Variable	Adolescents (*n* = 105)		Adults (*n* = 380)	
	Female	Male	Female	Male
	69	36	268	112
Marital status				
Unmarried	/		72	46
Married			185	61
Divorced/Widowed			11	5
ICD-11 diagnoses				
6A2 (psychotic disorders)	3	0	16	12
6A6 (bipolar disorders)	3	3	16	7
6A7 (depressive disorders)	54	21	100	37
6B (anxiety- or fear-related disorders)	3	7	120	49
6C2 (somatic symptom disorders)	0	1	15	6
others	6	4	1	1
PHQ-9 score				
<5	3	2	21	11
5-9	6	3	33	19
10-14	6	6	40	21
>= 15	54	25	174	61

All diagnoses are primary diagnoses based on ICD-11 criteria. Comorbid depressive symptoms were prevalent across groups (see **Table 2** for details).

In accordance with ICD-11, the patients were classified into the following diagnostic categories: 6A2 (psychotic disorders), 6A6 (bipolar disorders), 6A7 (depressive disorders), 6B (anxiety- or fear-related disorders), 6C2 (somatic symptom disorders), and other diagnoses (comprising one case of Alzheimer’s disease, one case of organic mental disorder, and ten cases of childhood mood disorders). Comorbidities were documented but not listed in **Table 1**. Notably, 50.3% of adults with primary anxiety/fear-related disorders (n=76/151) and 75.9% of adults with primary bipolar disorder (n=22/29) exhibited moderately severe (PHQ-9 15-19) or severe depressive symptoms (PHQ-9 ≥20), consistent with high comorbidity of depressive symptomatology across diagnostic categories.

According to PHQ-9 scores, patients were grouped based on the cutoff scores recommended by prior research: scores below 5 indicated no depression, 5–9 indicated mild depression, 10–14 indicated moderate depression, and 15 or higher indicated severe depression (4).

The vast majority of patients (92.4%) presented with mild to severe depressive symptoms. A substantial proportion exhibited severe depressive symptoms, with 64.7% of the total sample (*n* = 314) scoring 15 or higher on the PHQ-9—significantly exceeding the 225 patients diagnosed with 6A7 (depressive disorders) based on ICD-11 criteria.

The overall mean PHQ-9 score was 17.22 (*SD* = 7.00). An independent samples t-test revealed no significant gender difference, *t* (267.46) = -1.24, *p* = .215, with females scoring an average of 17.49 (*SD* = 6.87) and males 16.61 *(SD* = 7.26). Thus, gender was not used as a grouping variable in subsequent analyses. However, another independent samples t-test revealed a significant difference between adolescents (age < 16) and adults (age ≥ 16), *t* (177.05) = 3.15, *p* = .002. The mean PHQ-9 score was 19.03 for adolescents and 16.72 for adults.

Cross-tabulation of PHQ-9 scores and ICD-11 diagnoses (**Table 2**) revealed that patients diagnosed with ICD-11 6A7 (depressive disorders; n = 212) accounted for 43.7% of the total sample. All patients in this diagnostic subgroup scored ≥5 on the PHQ-9, with the majority (89.2%) attaining scores of ≥15. Anxiety- or fear-related disorders (n = 151) were the second most prevalent diagnosis, accounting for 31.1% of the sample. Although PHQ-9 scores among these patients were relatively evenly distributed, 50.3% (n = 76) still presented with major or severe depressive symptoms (PHQ-9 ≥ 15). In the subgroup of patients with bipolar disorder (n = 29), 75.9% (n = 22) exhibited severe or more profound depressive symptoms. Among the total sample in this study, the standard severity sub-bands of the PHQ-9 were as follows: 15–19 points (moderately severe, 112/485, 23.1%) and 20–27 points (severe, 218/485, 44.9%).

**Table 2:** PHQ-9 severity × ICD-11 diagnosis.

PHQ-9 score	<5	5-9	10-14	15-19	>= 20	Total
6A2 (psychotic disorders)	5	11	2	7	6	31
6A6 (bipolar disorders)	0	2	5	4	18	29
6A7 (depressive disorders)	0	7	16	57	132	212
6A8 (childhood mood disorders)	0	0	0	1	9	10
6B2 (obsessive-compulsive disorder)	0	1	1	1	2	5
6B4 (stress-related disorder)	1	1	3	1	4	10
6B6 (dissociative disorders)	3	5	1	2	1	12
6B8 (feeding or eating disorders)	0	1	0	0	0	1
6B (anxiety- or fear-related disorders)	15	22	38	37	39	151
6C2 (somatic symptom disorders)	2	5	8	2	5	22
others	0	0	0	0	2	2
Total	26	55	74	112	218	485

### 3.2 Reliability and validity of the PHQ-9

For the 485 valid patients (105 adolescents, 380 adults), the PHQ-9 demonstrated good internal consistency: α = 0.876 for adolescents and α = 0.883 for adults. Based on prior studies (e.g., 8, 18), two confirmatory factor analysis (CFA) models were specified: Model A, a unidimensional model, and Model B, a two-factor model comprising a cognitive-affective factor (items 1, 2, 6, 9) and a somatic factor (items 3, 4, 5, 7, 8). CFA was performed separately for adolescent and adult groups, with outcomes detailed in **Tables 3**, **4**.

**Table 3:** CFA results for adolescents.

Model	*χ*2/df	RMSEA	GFI	CFI	NFI	IFI	TLI
Model A	2.27	.110	.89	.91	.86	.92	.89
Model B	2.32	.112	.89	.91	.86	.92	.88

**Table 4:** CFA results for adults.

Model	χ2/df	RMSEA	GFI	CFI	NFI	IFI	TLI
Model A	4.11	.090	.94	.94	.92	.94	.92
Model B	3.86	.089	.94	.95	.93	.95	.93

In the adolescent group, both the unidimensional model (Model A) and two-factor model (Model B) demonstrated suboptimal RMSEA values (.110 and.112, respectively), which may partially reflect the small sample size (n=105)—a known factor that can falsely inflate RMSEA (30–32). Other fit indices (CFI = .91, GFI = .89) were marginally acceptable, indicating the models captured key variance in depressive symptoms despite potential RMSEA overestimation. These findings indicate that neither model adequately captured the latent symptom structure in this adolescent inpatient sample, potentially due to diagnostic heterogeneity within the sample or developmental variability in symptom presentation (33).

In contrast, both models demonstrated acceptable fit in the adult sample. The two-factor model (Model B) exhibited a slightly better fit than the unidimensional model (Model A), as evidenced by lower RMSEA values (.089 vs.090) and higher CFI values (.95 vs.94). Although RMSEA values for both models were slightly above the ideal threshold of.08, they fell within the range of marginally acceptable fit (32).

**Figure 1:**
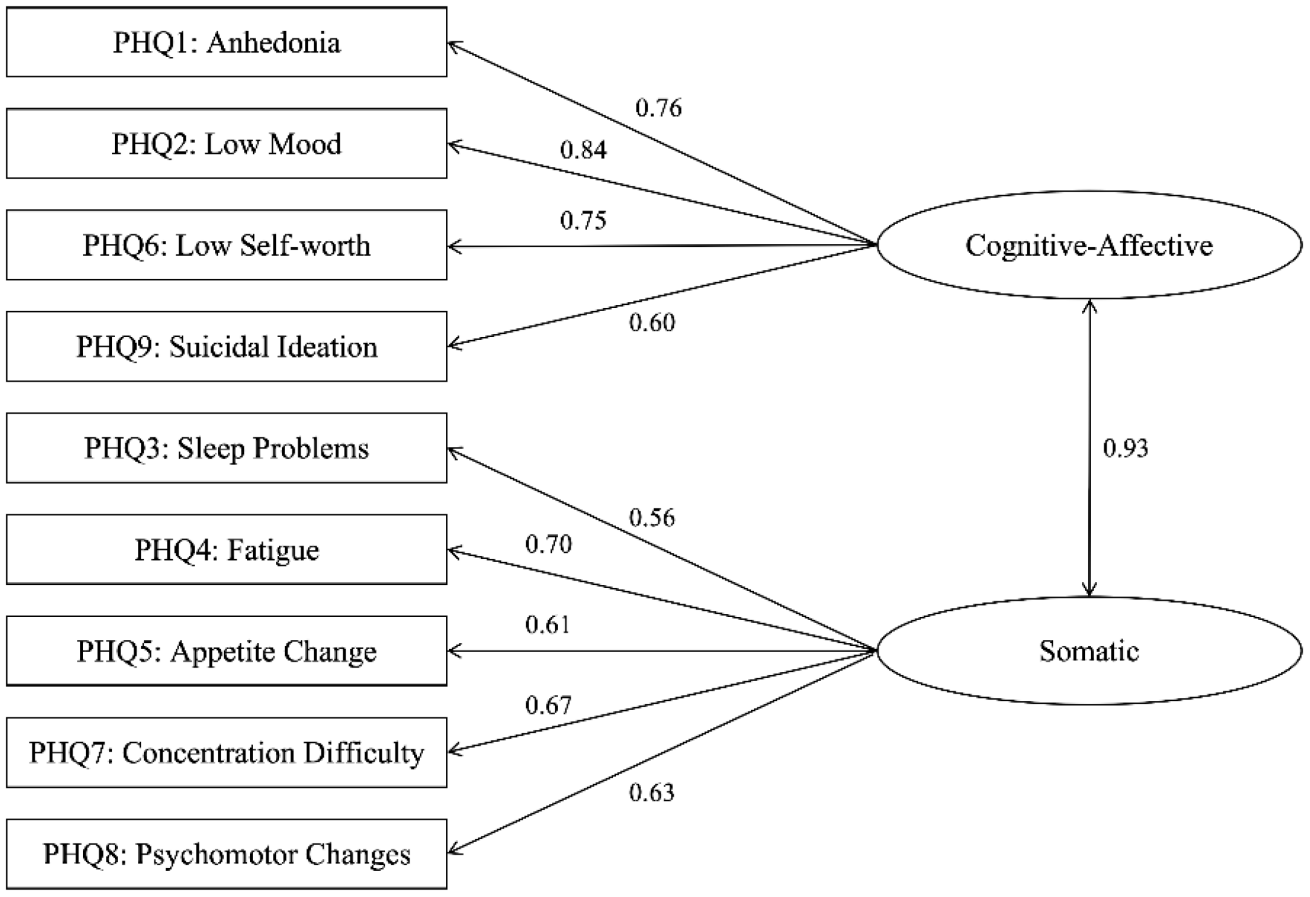
Two-factor model of the PHQ-9 in adult psychiatric inpatients.

The Satorra-Bentler scaled chi-square difference test was employed to compare nested models within each group. For the adolescent sample, no significant difference in model fit was observed between the two models (Δχ²(1) = 0.79, p = .374). In contrast, in the adult sample, the two-factor hierarchical model (Model B) provided a significantly better fit than the unidimensional model (Δχ²(1) = 6.49, *p* = .011), indicating a more differentiated structure of depressive symptoms in adults. Results are presented in **Table 5**. **Figure 1** depicts the standardized path diagram of the PHQ-9 in the adult psychiatric inpatient sample, elucidating the factorial structure and magnitude of item loadings.

**Table 5:** CFA model comparison.

Sample	Model	Df	AIC	BIC	χ²	Δχ²	ΔDf	P-value
Adolescents	Model A	27	2337.2	2385	61.24			
	Model B	26	2338.2	2388.7	60.26	0.79	1	0.374
Adults	Model A	27	9068.7	9139.6	110.94			
	Model B	26	9060.0	9134.9	100.32	6.49	1	0.011*

*p <.05.

### 3.3 Network analysis

#### 3.3.1 Network structure analysis

**Figure 2:**
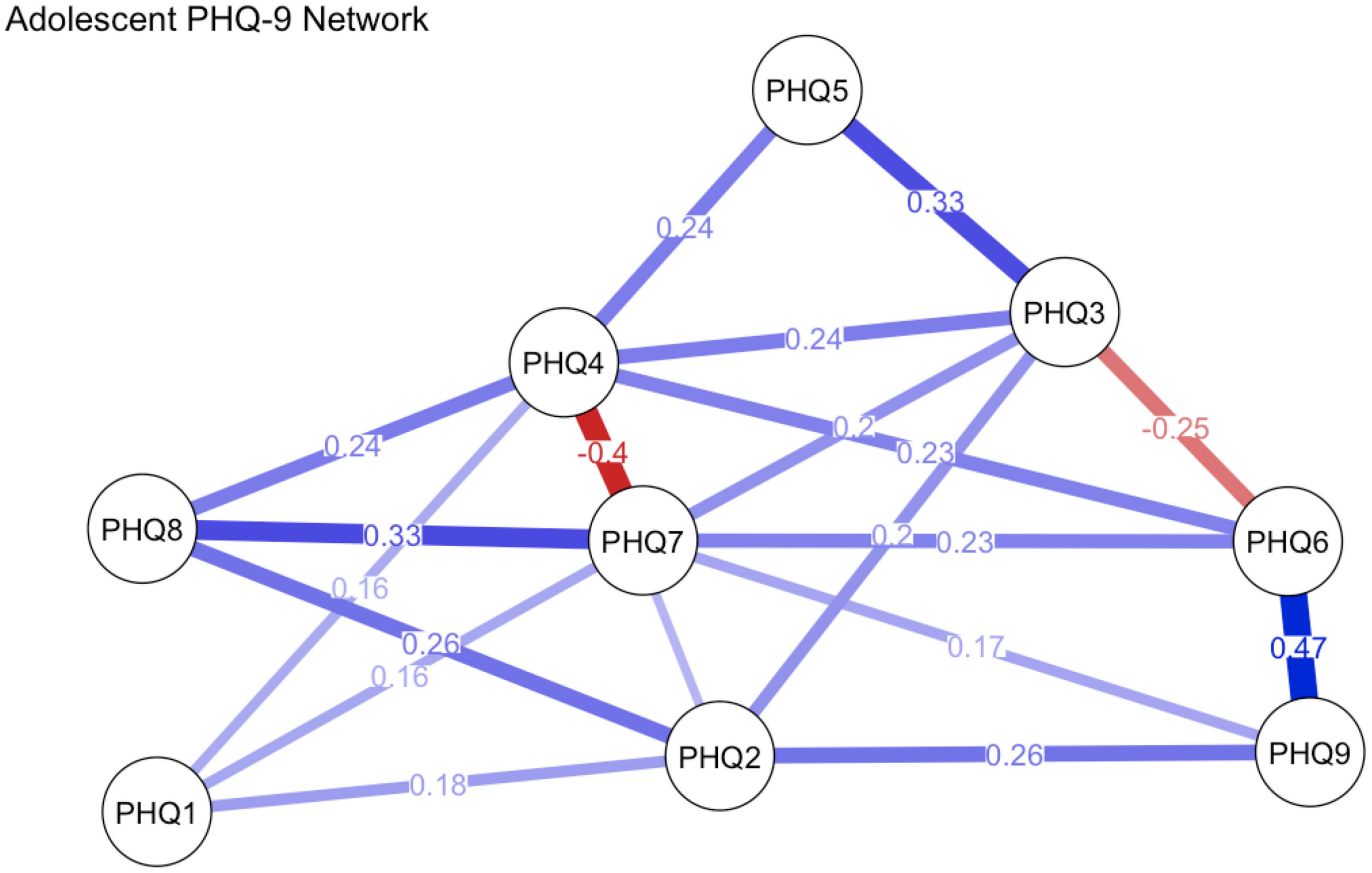
PHQ-9 network in adolescent sample.

**Figure 3:**
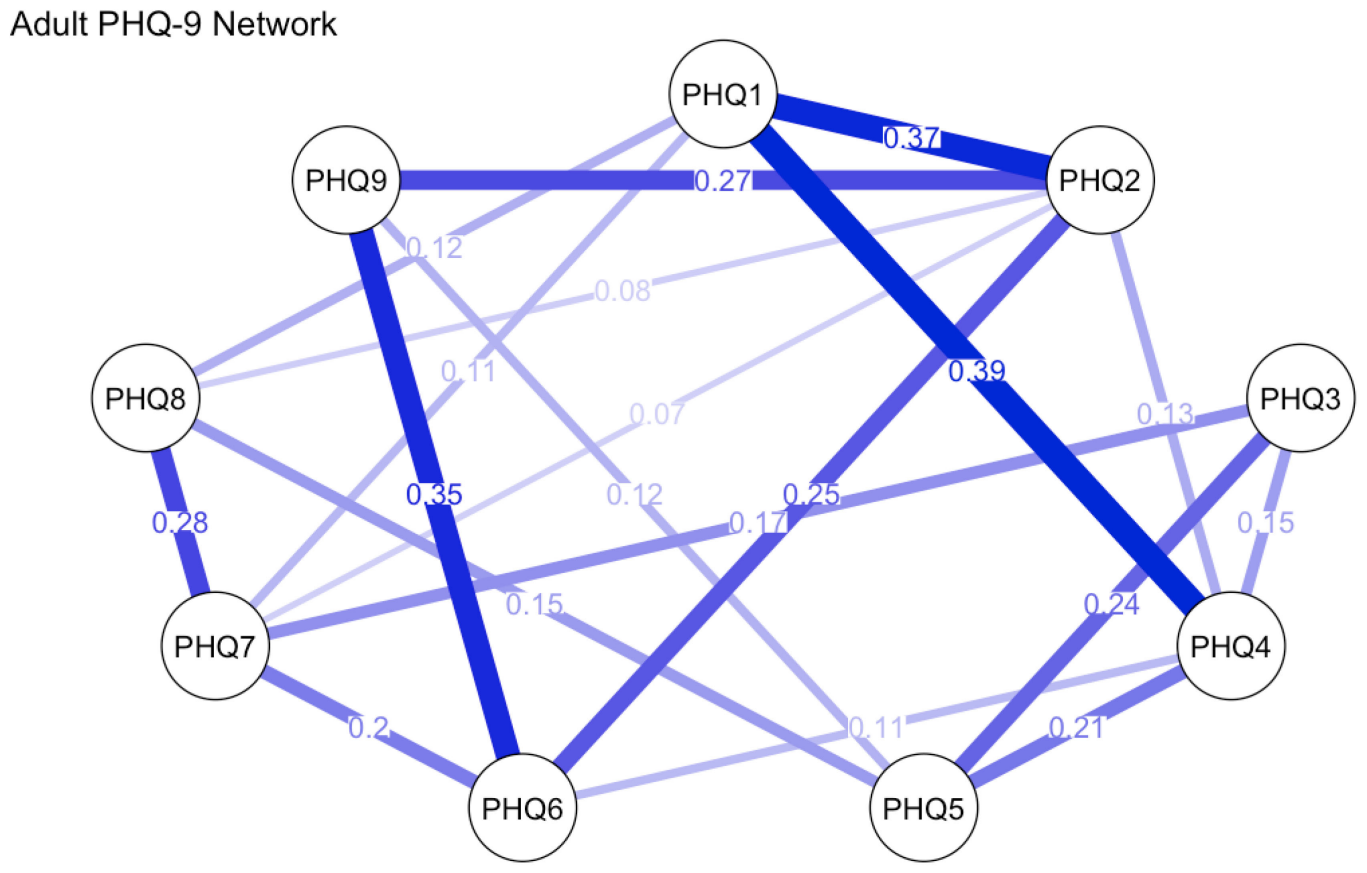
PHQ-9 network in adult sample.

Univariate normality tests were performed for each PHQ-9 item in both adolescent and adult groups. Findings showed all items adhered to normality across both age cohorts. Redundancy analysis was conducted using the goldbricker function from the network tools package in R to identify potentially overlapping items within the PHQ-9 across samples, treating all items as ordinal variables. In the adolescent sample, six item pairs were flagged as potential redundancies based on high inter-item correlations (r ≥ 0.5) and <25% significantly different correlations with remaining items: PHQ5 & PHQ1 (r = 0.51), PHQ5 & PHQ4 (r = 0.57), PHQ2 & PHQ1 (r = 0.59), PHQ4 & PHQ1 (r = 0.55), PHQ4 & PHQ2 (r = 0.56), and PHQ9 & PHQ6 (r = 0.64). No redundant item pairs met these criteria in the adult sample, suggesting clearer conceptual differentiation of items in this group. Given the moderate correlation levels, conceptual non-overlap of flagged items, and the need to maintain item-level comparability for subsequent network analyses, no item reduction was implemented. All nine PHQ-9 items were retained for network estimation, with analyses conducted using the estimate Network function in R. Network analysis results are presented in **Figures 2**, **3**.

In the adolescent group, the estimated network contained 38 nonzero edges, with an average edge weight of 0.177, indicating a moderately connected symptom structure. The strongest associations were found between item 6 (“Feeling bad about yourself or that you are a failure”) and item 9 (“Thoughts that you would be better off dead or hurting yourself”; edge weight = 0.467), item 7 (“Trouble concentrating”) and item 8 (“Moving or speaking slowly or being restless”; 0.330), and item 3 (“Trouble falling or staying asleep, or sleeping too much”) and item 5 (“Feeling tired or having little energy”; 0.328). These strong edges reflect meaningful clusters among cognitive, somatic, and self-esteem-related symptoms. In terms of node centrality, item 4 (“Feeling tired or having little energy”) demonstrated the highest strength (1.633), followed by item 7 (1.488), item 3 (1.221), and item 6 (1.180). This suggests that fatigue, concentration problems, sleep disturbances, and self-critical thoughts play especially influential roles in the depressive symptomatology of adolescents.

In the adult group, the estimated network similarly consisted of 38 nonzero edges, featuring a slightly elevated average edge weight of 0.197. The strongest connections were identified between item 1 (“Little interest or pleasure in doing things”) and item 4 (“Feeling tired or having little energy”) (edge weight = 0.387), item 1 and item 2 (“Feeling down, depressed, or hopeless”) (0.366), as well as item 6 (“Feeling bad about yourself”) and item 9 (“Thoughts of death or self-harm”) (0.349). These edges underscore prominent linkages between core affective symptoms and self-worth constructs. In terms of node centrality, item 2 (“Feeling down, depressed, or hopeless”) emerged as the most central symptom (strength = 1.173), followed by item 1 (0.984), item 4 (0.975), and item 6 (0.907). Collectively, these results highlight the central roles of low mood, anhedonia, fatigue, and negative self-evaluation in adult depressive presentations. **Table 6** displays the centrality indices of the nodes.

**Table 6:** Centrality indices of PHQ-9 items in adolescents and adults.

Node	Adolescent				Adult			
	Strength	Closeness	Betweenness	EI	Strength	Closeness	Betweenness	EI
PHQ1	0.50	0.01	0	0.50	0.98	0.02	16	0.98
PHQ2	1.03	0.02	6	1.03	1.17	0.02	8	1.17
PHQ3	1.22	0.02	6	0.71	0.55	0.01	0	0.55
PHQ4	1.63	0.03	6	0.84	0.98	0.02	8	0.98
PHQ5	0.57	0.02	0	0.57	0.71	0.02	2	0.71
PHQ6	1.18	0.02	6	0.67	0.91	0.02	6	0.91
PHQ7	1.49	0.02	8	0.70	0.83	0.02	8	0.83
PHQ8	0.83	0.02	2	0.83	0.63	0.01	2	0.63
PHQ9	0.89	0.02	2	0.89	0.74	0.02	2	0.74

#### 3.3.2 Stability and accuracy of the networks

We evaluated the stability of the estimated networks using case-dropping subset bootstrapping implemented in the *bootnet* package. The correlation stability coefficient (CS-coefficient) was used to quantify the proportion of the sample that could be omitted while maintaining a correlation of at least 0.7 with the original centrality metrics in ≥95% of bootstrap samples. For the adolescent network, the CS-coefficient for strength centrality was 0, indicating extremely poor stability. The edge weight CS-coefficient was 0.124, further confirming limited robustness. These findings suggest the adolescent network lacks sufficient stability for confident interpretation of node strength and edge comparisons. In contrast, the adult network demonstrated substantially better stability, with a strength centrality CS-coefficient of 0.361 and edge weight CS-coefficient of 0.516. These values fall within the acceptable to good range, supporting the robustness of centrality estimates in the adult network.

**Figure 4:**
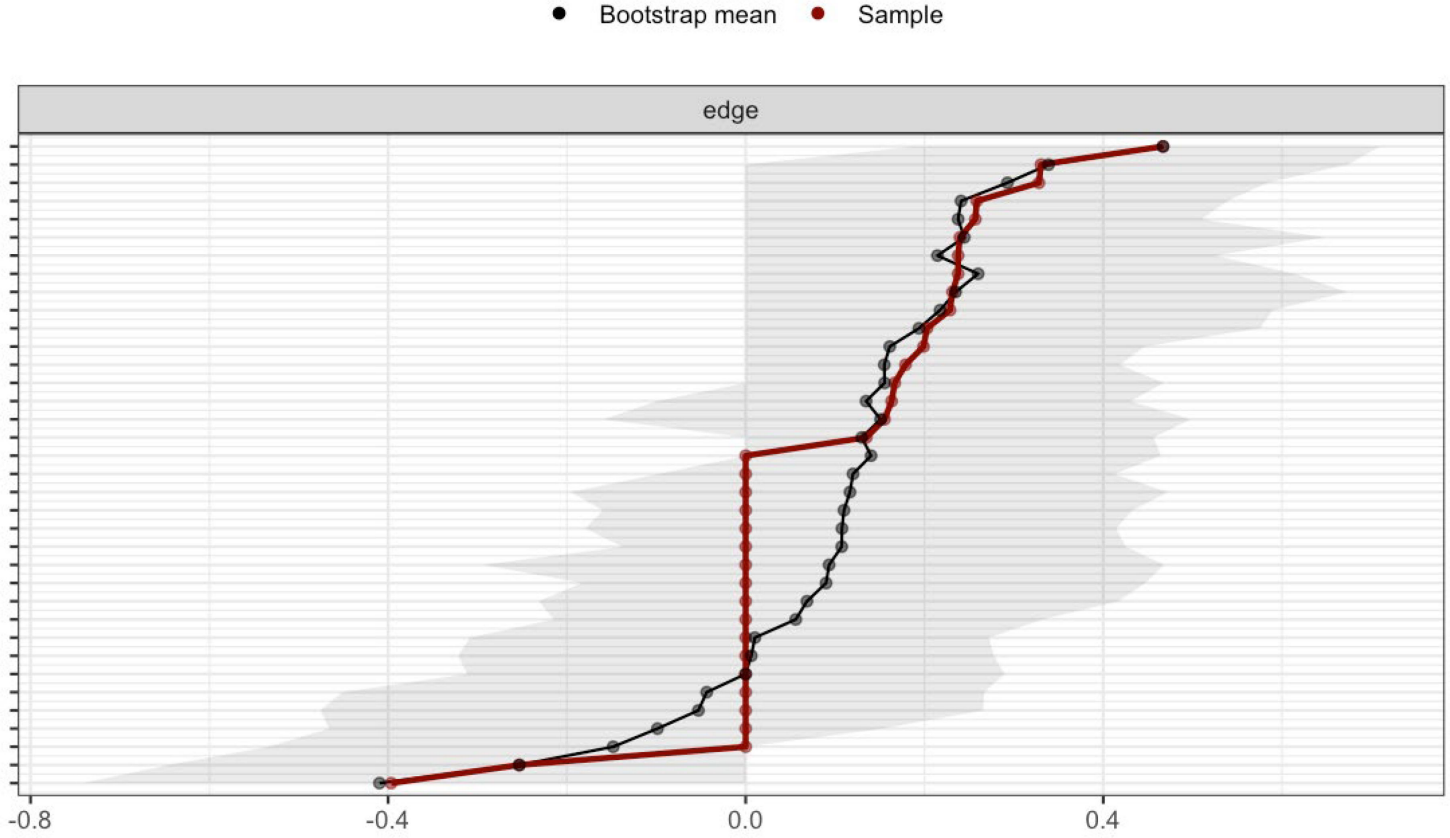
Edge weight CIs of adolescent network.

**Figure 5:**
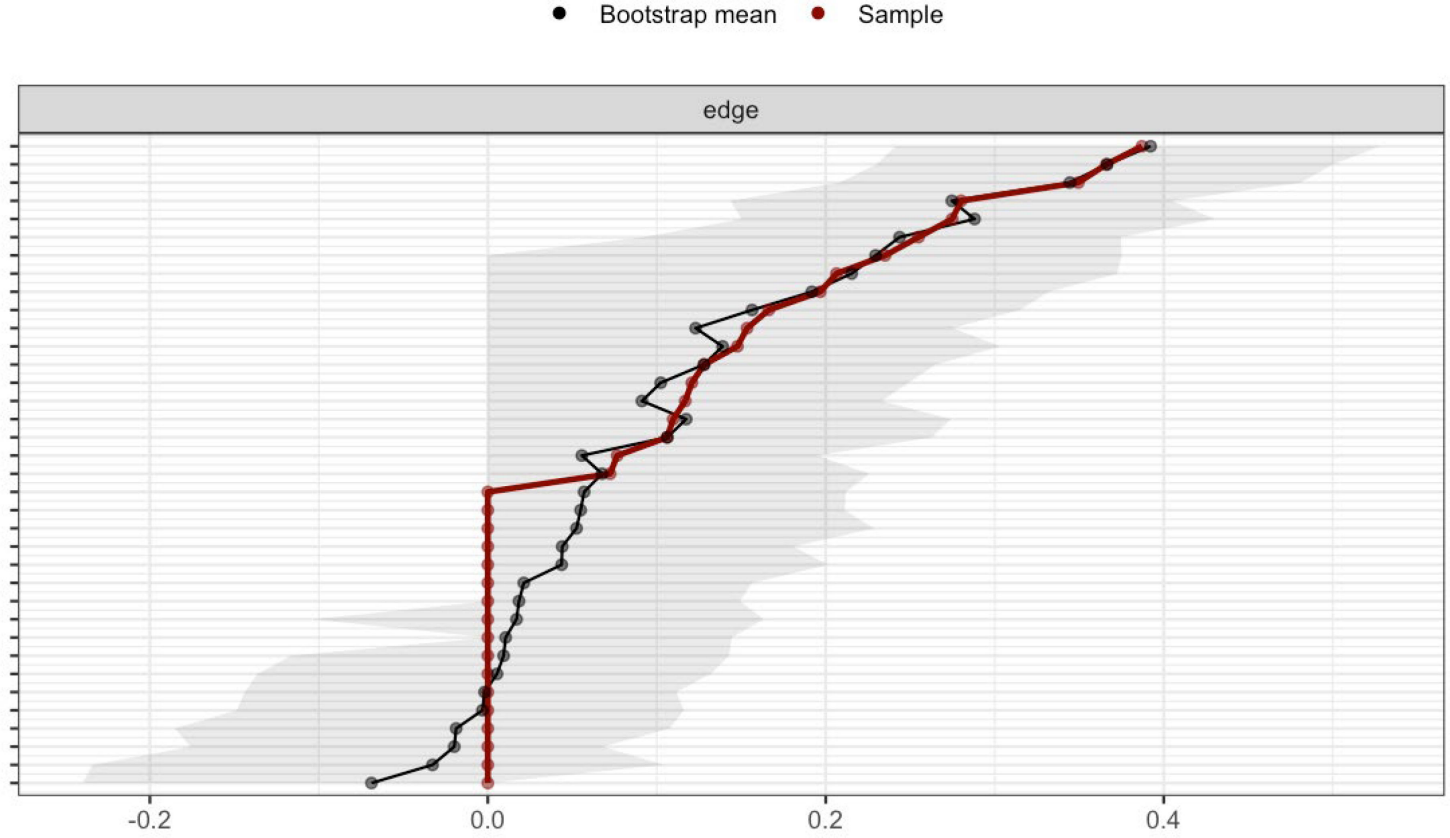
Edge weight CIs of adult network.

The stability of edge weights was evaluated via nonparametric bootstrapping. As illustrated in **Figures 4**, **5**, the width of the 95% confidence intervals (CIs) demonstrated substantial group-level variation, with narrower CIs observed in the adult network. This finding suggests higher stability of edge weights in adults compared to adolescents.

#### 3.3.3 Network comparison between adolescents and adults

**Figure 6:**
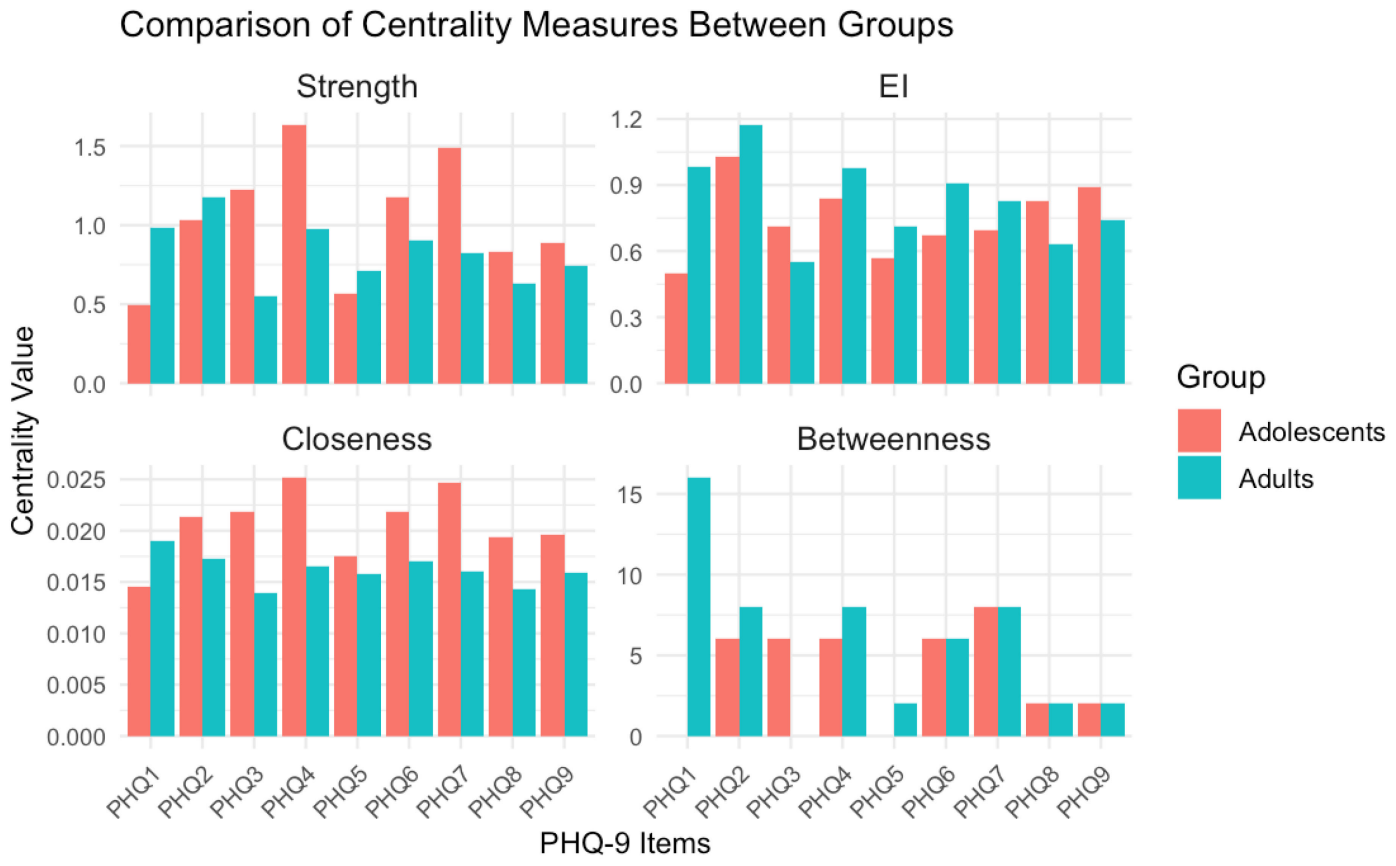
Comparison of centrality measures between groups. Adolescent network stability is poor (CS-coefficient=0), so centrality differences should be interpreted cautiously.

To examine the relative importance of PHQ-9 items within and across networks, we first compared four centrality indices—strength, expected influence (EI), closeness, and betweenness—between the adolescent and adult groups (see **Figure 6**).

Network stability analyses revealed notable differences between the two age groups: the adolescent network demonstrated poor stability (CS-coefficient = 0), indicating that both its edge weights and centrality estimates are unreliable. Notably, key symptoms such as anhedonia and fatigue exhibited weak centrality in this group, further constraining interpretability. In contrast, the adult network showed good stability (CS-coefficient = 0.72), with anhedonia (item 2) and depressed mood (item 1) emerging as central nodes.

The Network Comparison Test (NCT) found no significant differences in network structure (M = 0.211, p=0.598) or global strength (S = 0.262, p=0.186) between adolescents and adults. However, the poor stability of the adolescent network limits interpretation of these results. The null findings should be viewed with caution, as they may reflect measurement noise rather than true age invariance.

### 3.4 ROC analysis

**Figure 7:**
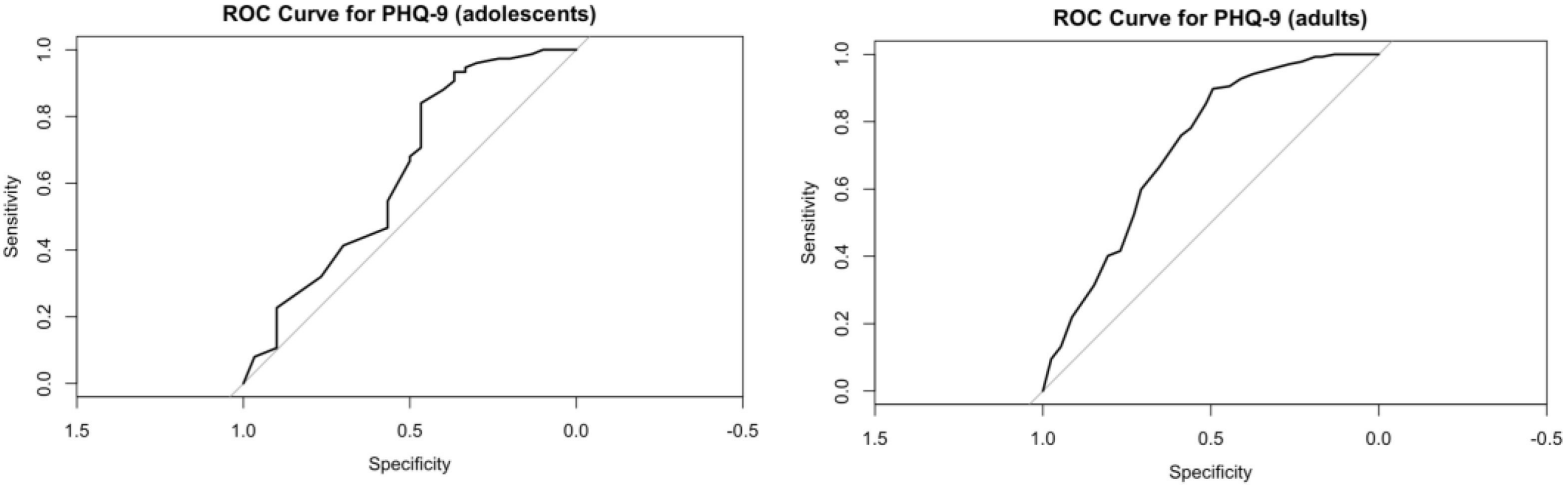
ROC curve for PHQ-9 in diagnosing depression.

For both adolescents and adults, receiver operating characteristic (ROC) analysis was performed using the ICD-11 diagnosis of “6A7 Depressive Disorder” as the reference standard to evaluate the PHQ-9’s performance in identifying depressive disorders. The adolescent group demonstrated an area under the curve (AUC) of 0.636. Threshold analysis revealed that a cut-off score of 15.5 yielded a sensitivity of 0.84, specificity of 0.47, and a corresponding Youden index of 0.31. In contrast, the adult group showed a higher AUC of 0.724. Threshold analysis indicated that a cut-off score of 14.5 achieved a sensitivity of 0.90, specificity of 0.49, and a Youden index of 0.39. The higher Youden index in adults suggests relatively superior diagnostic efficiency of the PHQ-9 in this subgroup (see **Figure 7**).

## 4 Discussion

This study systematically evaluated the psychometric properties of the PHQ-9 among adolescent and adult psychiatric inpatients in China, with a specific focus on its factorial structure, symptom network characteristics, and optimal diagnostic threshold determination. The findings revealed both cross-age-group consistencies and developmentally relevant divergences, providing critical insights into the scale’s clinical applicability and construct validity within this specialized population.

Confirmatory factor analysis (CFA) results revealed that the two-factor model—comprising cognitive-affective and somatic dimensions—exhibited superior model fit compared to the unidimensional model in adults (Δχ²=6.49, p=0.011). In adolescents, however, no significant difference in fit was observed between the two models (Δχ²=0.79, p = 0.374), with both models showing marginally acceptable fit (CFI = 0.91) despite inflated RMSEA (likely due to small sample size; 32). Beyond the potential influence of a small sample size, this finding may also reflect greater heterogeneity in symptom presentation or cognitive immaturity in how adolescents endorse symptoms - an observation that aligns with developmental research. This literature highlights that adults typically conceptualize depressive symptoms through distinct cognitive and somatic frameworks, whereas adolescents often exhibit a more diffuse, less differentiated symptom profile (15).

Building on recent work documenting heterogeneity in adolescent emotional dysregulation (24, 27), we propose that adolescent depressive symptoms constitute an undifferentiated syndrome - one that may be more complex than its adult equivalent. This syndrome is embedded within the broader context of emotional susceptibility differentiation: a developmental process intertwined with biological factors, which manifests in the trajectory of personality development (encompassing both pathological and normative domains; 34).

Network analysis further unveiled age-dependent variations in symptom architecture. Although both networks comprised 38 nonzero edges, the adult network exhibited marginally stronger connectivity (mean edge weight = 0.197 vs. 0.177). Centrality analyses revealed that low mood (PHQ2) and anhedonia (PHQ1) served as core symptoms in adults, aligning with prior evidence highlighting affective disturbances as the clinical pivot of adult depression (8). By contrast, adolescents manifested a distinct structural profile, with fatigue (PHQ4) and concentration difficulties (PHQ7) emerging as central nodes. These findings may reflect the somatic presentation of adolescent depression—a pattern commonly documented in East Asian populations and congruent with somatization theory, which posits that emotional distress in this demographic is often expressed via physical complaints (35).

Significantly, the adolescent network demonstrated poor stability (CS-coefficient for strength = 0), thereby limiting the reliability of centrality estimations. This observation is likely attributable to the smaller sample size (n = 105) and heightened symptom heterogeneity. Future investigations should prioritize replication in larger cohorts to validate these findings. Edge-level analyses unveiled both shared and age-specific symptom associations. For example, the robust linkage between low self-worth (PHQ6) and suicidal ideation (PHQ9) across age groups underscores the clinical salience of self-evaluation in suicidal risk assessment. By contrast, adolescents exhibited stronger modularity among sleep disturbance (PHQ3), fatigue (PHQ4), and concentration difficulties (PHQ7), whereas adults showed tighter affective-fatigue symptom couplings. These topological differences may inform the design of age-tailored interventions, targeting symptom clusters that drive depressive severity propagation.

ROC analysis furnished additional evidence of age-dependent diagnostic disparities in PHQ-9 performance. Although both adolescent and adult inpatient groups exhibited acceptable levels of discriminative validity, adult participants demonstrated superior overall accuracy (AUC = 0.724 vs. 0.636 for adolescents) and a higher Youden index (0.39 vs. 0.31 for adolescents). Notably, the age-specific optimal diagnostic thresholds identified in this study—15.5 for adolescents and 14.5 for adults—surpassed the internationally recommended PHQ-9 cutoff of 10 (4, 6). This elevation in optimal thresholds may be attributed to three key factors: (1) the heightened clinical severity inherent to psychiatric inpatient populations (distinct from community samples); (2) cultural factors, such as the amplification of somatic symptoms observed in East Asian populations (35); and (3) the high comorbidity of depressive symptoms with non-depressive primary diagnoses (see **Table 2**; 64.7% of participants scored ≥15 on the PHQ-9, compared to only 43.7% who received a diagnosis of primary depressive disorder). Further support for the role of comorbidity comes from the relatively low specificity values observed in both groups (adolescents: 0.47; adults: 0.49), as comorbid depressive symptoms may increase false-positive classifications for primary depressive disorder (ICD-11 6A7).

Consistent with these ROC-derived findings, we argue that the conventional PHQ-9 cutoff of 10—while classifying 92.4% of our psychiatric inpatient sample as “depressed”—fails to distinguish between patients with primary depressive disorder (ICD-11 6A7) and those with depressive symptoms comorbid with other psychiatric conditions. In contrast, the higher age-specific cutoffs (15.5 for adolescents, 14.5 for adults) proposed in this study more effectively target patients with clinically significant depression. By reducing the over inclusion of subthreshold depressive cases (i.e., individuals with mild or transient symptoms not meeting diagnostic criteria for primary depression), these cutoffs correct for the severity underestimation that occurs in the true primary depressive disorder subgroup within Chinese psychiatric inpatient samples. Collectively, these findings establish an empirical foundation for optimizing age-stratified depression screening protocols in Chinese psychiatric inpatients. The higher cutoffs (15.5, 14.5) are not intended to replace the conventional cutoff of 10 for community samples (11) but rather to address the unique severity profile of inpatient populations, where comorbid and subthreshold symptoms are common.

Several study limitations merit acknowledgment. First, the cross-sectional nature of the design restricts causal inference regarding temporal symptom dynamics. Second, the small adolescent sample (n=105) may have inflated RMSEA values in CFA, overstating model misfit. Future studies with larger adolescent samples should validate the PHQ-9’s factor structure in this group. The adolescent network’s poor stability (CS-coefficient=0) invalidates confident comparisons with the adult network. Future studies with larger adolescent samples are needed to clarify age-related differences in symptom network structure. Third, recruitment from a single psychiatric hospital may introduce selection bias, potentially compromising external validity to community populations. Fourth, a potential limitation is the uneven sample size (adolescents: n=105; adults: n=380) and differing primary diagnostic distributions between age groups. Although comorbid depressive symptoms were prevalent across all diagnostic categories (ensuring comparability of the PHQ-9’s target construct), future studies with larger, matched adolescent samples (e.g., stratified by primary diagnosis) would strengthen causal inferences about age-related differences. The last limitation is that the PHQ-9 was not originally designed for children <12, so results for this subgroup should be interpreted cautiously.

In summary, the PHQ-9 exhibits good reliability for assessing depressive symptoms among Chinese psychiatric inpatients, including both adolescent and adult subgroups. However, its latent structure and optimal diagnostic cutoffs differ by age: the conventional PHQ-9 cutoff of 10 fails to align with the unique severity profile of this inpatient population, as it cannot distinguish between patients with primary depressive disorder (ICD-11 6A7) and those presenting with comorbid or subthreshold depressive symptoms—leading specifically to the misclassification of symptom severity (rather than the overestimation of depression prevalence). Age-specific cutoffs (15.5 for adolescents, 14.5 for adults) are recommended exclusively for Chinese psychiatric inpatients and are not intended to replace the conventional cutoff of 10 for community samples; notably, the influence of comorbidity on the study’s findings should be accounted for when interpreting these results.

## Data Availability

The raw data supporting the conclusions of this article will be made available by the authors, without undue reservation.
